# The Beneficial Effects of Renal Transplantation on Altered Oxidative Status of ESRD Patients

**DOI:** 10.1155/2016/5757645

**Published:** 2016-07-28

**Authors:** José Ignacio Cerrillos-Gutiérrez, Alejandra Guillermina Miranda-Díaz, Priscila Preciado-Rojas, Benjamín Gómez-Navarro, Sonia Sifuentes-Franco, Sandra Carrillo-Ibarra, Jorge Andrade-Sierra, Enrique Rojas-Campos, Alfonso Martín Cueto-Manzano

**Affiliations:** ^1^Department of Nephrology and Transplants, Specialties Hospital, National Occidental Medical Centre, The Mexican Social Security Institute, Guadalajara, 44349 Guadalajara, JAL, Mexico; ^2^Department of Physiology, University Health Sciences Centre, The University of Guadalajara, Guadalajara, 44150 Guadalajara, JAL, Mexico; ^3^Kidney Diseases Medical Research Unit, Specialties Hospital, National Occidental Medical Centre, Mexican Social Security Institute, Guadalajara, 44349 Guadalajara, JAL, Mexico

## Abstract

Renal transplantation (RT), has been considered the best therapeutic option for end stage renal disease (ESRD).* Objective*. To determine the effect of RT on the evolution of oxidative DNA status.* Methods*. Prospective cohort (*N* = 50 receptors of RT); genotoxic damage, 8-hydroxy-2′-deoxyguanosine (8-OHdG), and DNA repair enzyme, human 8-oxoguanine-DNA-N- glycosylase-1 (hOGG1); and antioxidants, superoxide dismutase (SOD) and glutathione peroxidase (GPx), were evaluated.* Results*. Before RT, 8-OHdG were significantly elevated (11.04 ± 0.90 versus 4.73 ± 0.34 ng/mL) compared to healthy controls (*p* = 0.001), with normalization after 6 months of 4.78 ± 0.34 ng/mL (*p* < 0.001). The same phenomenon was observed with hOGG1 enzyme before RT with 2.14 ± 0.36 ng/mL (*p* = 0.01) and decreased significantly at the end of the study to 1.20 ng/mL (*p* < 0.001) but was higher than controls, 0.51 ± 0.07 ng/mL (*p* < 0.03). Antioxidant SOD was elevated at 24.09 ± 1.6 IU/mL versus healthy controls (*p* = 0.001) before RT; however, 6 months after RT it decreased significantly to 16.9 ± 1.6 IU/mL (*p* = 0.002), without achieving the levels of healthy controls (*p* = 0.01). The GPx, before RT, was significantly diminished with 24.09 ± 1.6 IU/mL versus healthy controls (39.0 ± 1.58) (*p* = 0.01), while, in the final results, levels increased significantly to 30.38 ± 3.16 IU/mL (*p* = 0.001).* Discussion*. Patients with ESRD have important oxidative damage before RT. The RT significantly reduces oxidative damage and partially regulates the antioxidant enzymes (SOD and GPx).

## 1. Introduction

End stage renal disease (ESRD) is a global, public health problem that causes great economic burden and increases morbidity and mortality [1]. ESRD is considered the last stage in chronic kidney disease (CKD), where uremia is the most serious complication. Renal replacement therapies (RRT) are needed to manage ESRD patients, among which are peritoneal dialysis (PD), hemodialysis (HD), and renal transplantation (RT) [2]. The RT is considered the treatment of choice and the best option for ESRD patients.

Oxidative stress (OS) results from imbalance between production of oxidants and the mechanisms of antioxidant defense [3]. In ESRD there is dysregulation of the oxidative state and the antioxidant systems due to the presence of inflammation, anemia, high levels of homocysteine, and treatment conditions as parenteral iron use, dialysate nonbiocompatible membranes, and significant reduction in endogenous antioxidants levels [4].

In addition, HD is considered an important source of oxidative stress in ESRD, due to the production of interleukins and anaphylatoxins (powerful activators of the nicotinamide adenine dinucleotide phosphate (NADPH) oxidase) during HD sessions. The enzyme NAPDH oxidase is responsible for the overproduction of reactive oxygen species (ROS) and constitutes the link between the activation of different cells types as leukocytes and organic toxicity. The HD can induce ROS production by many paths, one the bioincompatibility of dialysis system, the reactivity of dialysis membrane, and the production of endotoxins in the dialysate, and can induce deterioration of the antioxidant mechanisms. Superoxide anion (O_2_^−^) levels significantly increase after the HD session. Also, they produce high levels of homocysteine in plasma in the early phase of CKD promoting a prooxidative state by the interaction with hydrogen peroxide (H_2_O_2_) [5].

Antioxidant enzymes, superoxide dismutase (SOD), glutathione peroxidase (GPx), and catalase, are the main defense system against ROS and oxidative stress. During the oxidative phosphorylation of the mitochondria an electron is transferred to oxygen which results in formation of the O_2_^−^ that converts into hydrogen peroxide (H_2_O_2_) by the SOD, or it transforms into the hydroxyl radical. The O_2_^−^ is a highly reactive species capable to react with proteins, lipids, and nucleic acids [6]. There are three isoforms of SOD, SOD1 (CuZnSOD) which is present in red blood cells, SOD2 (MnSOD) in the mitochondria, and SOD3 in the extracellular media [7]. GPx is responsible for the conversion of H_2_O_2_ and other organic peroxides to water and oxygen [8]. Five isoforms of GPx have been identified; two are present in human red blood cells: GPx 1 [9] and GPx 3 (eGPx), which are produced by the kidney, in plasma [10].

Peritoneal dialysis uses peritoneum as “natural” dialysis membrane to eliminate the waste products from blood flow. The success and efficacy of PD depend on the integrity of the peritoneal membrane; PD itself produces chronic inflammatory conditions in the peritoneal cavity that coincide with increased levels of proinflammatory cytokines, which alter the integrity of the tissue (peritoneal membrane). The high concentrations of glucose and glucose metabolites in PD solutions promotes structural and functional reorganization of the peritoneal tissue in the long term [11]; this condition is associated with an important increase in malondialdehyde production and a decrease of antioxidants compared to healthy controls [12].

Renal transplant seems to induce less oxidative stress compared to patients routinely dialysed. However, in RT other factors can induce OS, for example, immune response to allograft, ischemia/reperfusion injury, infections, and immunosuppressive therapy [13]. Inflammation and OS can produce graft tissue damage due to the formation of fibrosis and nephrons loss by necrosis or apoptosis [14].

When the O_2_^−^ levels increase, can be converted to H_2_O_2_ by the SOD enzyme, or transform into the hydroxyl radical (OH^−^) [15], this is highly reactive and capable of reacting with the nitrogen-base guanine in the DNA [16]. The 8-hydroxy-2′-deoxyguanosine (8-OHdG) is the product of oxidative DNA damage, and it is a sensitive and specific marker with higher mutagenic effect [17, 18]. In human cells oxidative damage is primarily repaired by the endonuclease enzyme, 8-oxoguanine-DNA-N-glycosylase- 1 (hOGG1), through mechanisms of base excision [19].

Renal transplant is considered the best therapeutic option in patients with ESRD, with higher survival and quality of life compared to HD and PD patients; this RRT is associated with higher OS [20]; however, there is scarce information regarding OS after RT. The aim of this study was to evaluate the beneficial effect of RT on the evolution of oxidative* status*.

## 2. Material and Methods

A prospective cohort, with 50 patients subjected to RT with a six-month follow-up, was performed; all patients were from the Division of Nephrology/Organ Transplants at Unidad Médica de Alta Especialidad, UMAE, of Mexican Social Security Institute, in Guadalajara, Jalisco, Mexico. Receptors of a first RT from living donor (related or unrelated) were included, between 16 and 50 years of age, who agreed to participate in the study (signing an informed consent form). Patients with diabetes mellitus and end stage renal disease due to inflammatory disease (vasculitis, systemic lupus erythematous, or other connective tissue diseases or intestinal inflammatory illnesses) were excluded. Preemptive RT or multiple organ recipients were also excluded. All HD patients (18) receive 3 h/3 sessions per week with a similar conventional HD treatment; of them, 28% had fistulae and the remaining 72% had permanent HD catheter; they use polysulphone hemofilter (no re-used); all HD prescriptions were individualized by their own clinicians, according to their clinical conditions as ultrafiltration rate,* Kt/V*, and so forth.

Exclusion criteria were as follows: loss of renal graft function within the follow-up period, presence of severe systemic infection or infection due to cytomegalovirus at the time of the follow-up evaluations, and severe acute rejection treated with high doses of steroids or thymoglobulin. All patients receive the same triple immunosuppressive scheme based on the following: prednisone, mofetil mycophenolate, and tacrolimus; only tacrolimus had to be adjusted according to serum levels by their clinicians.

All oxidative markers were evaluated baseline and follow-up (6 months), using ELISA to determine DNA damage with 8-OHdG and the repairing enzyme hOGG1; and endogenous antioxidative markers SOD and GPx were evaluated with colorimetric techniques.

As a control of normal OS levels, 20 healthy subjects of similar age (36 years old) and gender (8 females and 12 males) were included as a control group, to standardize the normal values of reagents (blood donors from the blood bank who agreed to donate 10 mL extra blood apart from the amount donated).

Twenty-four h before RT 10 mL of venous blood was collected in a tube with 0.1% ethylenediaminetetraacetic acid (EDTA). The plasma was separated by centrifuge at 3000 revolutions per minute (rmp) for 10 minutes at room temperature, and the samples were stored at −80°C until processing.

### 2.1. 8-Hydroxy-2′-Deoxyguanosine

The manufacturer's suggested method for the ELISA kit was followed (8-hydroxy-2′-deoxyguanosine number ab10124 Abcam®, Cambridge, United Kingdom). The plasma sample, EIA buffer, the standards, and the 8-OHdG-AChE tracer were added to all the wells except the blank. Then the monoclonal antibody 8-OHdG was added and the plate was incubated for 18 h at 4°C. The plate was washed with the buffer for the recommended times and 200 *μ*L of Ellman's reagent was added to each well. The optical density was read at 405 nm.

### 2.2. 8-Oxoguanine-DNA-N-Glycosylase-1

Repair of the oxidative damage to DNA was determined through the use of a commercial kit (human 8-oxoguanine-DNA-N-glycosylase MBS702793, MyBiosource®, San Diego, CA). The manufacturer's instructions were followed, and the reactive species and samples were prepared for the indicated dilutions. 100 *μ*L of plasma and standards were added to the wells and the plate was incubated at 37°C. Then, the biotinylated antibody was added and incubated under the same conditions. The corresponding washings were done and the HRP-avidin was added, followed by the substrate and then the stop solution at the corresponding times. The optical density was read at 450 nm.

### 2.3. Superoxide Dismutase

The instructions of the kit manufacturer were followed (SOD number 706002, Cayman Chemical Company®, USA) for the detection of O_2_^−^ generated by the xanthine oxidase and hypoxanthine enzymes through the reaction of tetrazolium salts. The serum samples were diluted 1 : 5 in sample buffer: 200 *μ*L of the radicals' detector, diluted 1 : 400, was placed, and 10 *μ*L of the sample was then added. After slow agitation, 20 *μ*L of xanthine oxidase was added to the wells. The microplate was incubated for 20 minutes at room temperature and the absorbency was read at a wavelength of 440 nm. The levels are reported in IU/mL.

### 2.4. Glutathione Peroxidase

Measurement of GPx activity was performed according to the manufacturer's instructions (Bioxytech GPx-340, cat, 21017, OXIS Int, CA, USA). The reagent was based on the oxidation of reduced glutathione in the presence of tert-butyl hydroperoxide, glutathione reductase, and NADPH. The decrease in absorption at 340 nm following the substrate addition was recorded and a decreased rate of absorption is directly proportional to GPx activity.

For all of the technical readings of optical density the Synergy HT (BIOTEK) microplate reader was used.

### 2.5. Statistical Analysis

Results are expressed as mean ± SD to evaluate differences within the group, Wilcoxon's Signed Rank test was used; and to evaluate differences between groups Mann-Whitney* U* test was done. A value of *p* ≤ 0.05 was considered significant, with a confidence interval of 95%.

### 2.6. Ethics

The study was evaluated and approved by the Local Ethics and Research Committee at the* Unidad Medica de Alta Especialidad, Centro Médico Nacional de Occidente, IMSS* (R-2015-1301-91).

## 3. Results

Clinical and metabolic results are shown in Table 1. Seventy percent (35) were males; the average age was 31 years; height was 1.66 metres, and weight was 64 kg; two-thirds had PD as RRT; there were metabolic findings in hemoglobin, lipids creatinine, and albumin according to an ESRD patient; inflammation (determine by CRP) was present in at least 25%. At the end of the follow-up the low density cholesterol decreased significantly (LDL–*p* = 0.008) and the high density cholesterol increased significantly (HDL–*p* < 0.001). As expected, renal function improved significantly; and CRP decreases too.

### 3.1. 8-Hydroxy-2′-Deoxyguanosine

Normal plasma levels of the 8-OHdG marker were 4.7 ± 0.3 ng/mL. Evaluations made 24 h before RT in patients with ESRD were significantly elevated with 11.04 ± 0.9 ng/mL versus healthy controls (*p* = 0.001). However, the significant decrease between the measurement prior to RT and the measurement six months later is noteworthy: 4.7 ± 0.3 ng/mL, reaching normal limits (*p* < 0.001). This finding suggests RT low oxidative damage to DNA in patients with ESRD who undergo RT (Table 2).

### 3.2. 8-Oxoguanine-DNA-N-Glycosylase-1

Normal levels of the hOGG1 were 0.51 ± 0.07 ng/mL. However, measurements 24 h before patients underwent RT were importantly elevated at 2.14 ± 0.36 ng/mL (*p* = 0.01) compared to healthy controls, possibly in an attempt to counteract the effect of 8-OHdG. It is noteworthy that the final results of the enzyme decreased significantly to 1.20 ng/mL (*p* < 0.001) without reaching normal limits (*p* < 0.03) (Table 2).

### 3.3. Antioxidants

The SOD enzyme and the GPx are among the endogenous antioxidant enzymes that protect from oxidative damage. Normal levels of SOD found in healthy controls were 10.2 ± 0.09 IU/mL. Twenty-four h before RT the SOD was significantly increased with 24.09 ± 1.6 IU/mL versus healthy controls (*p* = 0.001). In the final results, the enzyme demonstrated a significant difference compared to the levels obtained in healthy controls, although it did not reach the normal limits (*p* = 0.01). On the other hand, the enzymatic activity of the GPx behaved differently. Levels in healthy controls were 39.0 ± 1.58 U/minute/mg of protein, and the initial levels in patients were significantly diminished with 24.14 ± 2.53 U/minute/mg of protein compared to the healthy controls. Six months after RT an increase was observed in the enzyme's activity with 30.38 ± 3.16 U/minute/mg of protein (*p* = 0.001) (Table 2).

## 4. Discussion

Patients with CKD frequently had inflammation and OS and can contribute to deterioration of renal function [21, 22]. The RRT could also increase inflammation and OS in different ways. In PD the human peritoneal mesothelial cells, a critical component of the peritoneal membrane, play an important role in the suitability of PD. The loss of these cells can contribute to the appearance of complications from PD due to the high levels of glucose dialysate, a key factor that favors the appearance of functional alterations and death of the human peritoneal mesothelial cells [23]. Patients subjected to HD show an increase oxidative damage to DNA and lower antioxidant activity [24]. In a study reported in 2006 performed in 7 patients with RT, ELISA was used to determine serum levels of 8-OHdG before reperfusion of the transplant, without an existing association between the serum levels of 8-OHdG just before the reperfusion and in the postoperative course. In all patients, serum 8-OHdG levels increased after reperfusion and decreased 2 h later; however, in 6 patients the same levels as the preoperative evaluation were maintained [25]. It has been reported that an increase in 8-OHdG (a product of oxidized DNA) is associated with resistance to erythropoietin and is found to be elevated in HD patients [26]. In the present study, a higher oxidative damage to the DNA was found, determined by higher levels of 8-OHdG (oxidant of the deoxyguanosine, base component of the DNA) 24 h before RT. However, the decrease to normal levels of 8-OHdG at the end of follow-up was evident, suggesting that RT is effective to reduce oxidative damage to the DNA.

Evaluation of specific mechanisms of DNA base excision can be done through determination of the glycosylase enzymes (hOGG1) that have specificity for 8-OHdG [27]. The hOGG1 can repair oxidative damage to the DNA, and it is well known that the OGG1 gene alteration increases susceptibility of the human cells to toxic compounds related to some toxins such as tobacco smoke, alcohol, and uremia, with a strong relationship between deregulation of the hOGG1 enzyme and the risk of suffering different kinds of cancers (e.g., laryngeal), due to consumption of tobacco and alcohol. This is the first study that evaluates the hOGG1 associated with uremia in ESRD patients and the evolution after RT [28].

On the other hand, we found dysregulation of the endogenous antioxidant enzymes (SOD and GPx). The baseline SOD levels were significantly elevated and normalized at the 6-month follow-up. This can be explained as a compensatory response to the important OS in which these patients were before RT, as previously reported in other chronic degenerative pathologies [29]. Recently, it was published that the plasma SOD was significantly elevated in patients subjected to HD before RT compared to healthy controls, with subsequent decrease in the endogenous concentrations after RT [30], similar to the findings of our study. The GPx activity was importantly diminished compared to healthy controls and significantly increases 6 months after RT. This finding could be explained by the increase in SOD, since the SOD is the first antioxidant enzyme to act in the presence of oxidative damage [31]. In general we found an improvement of other markers of oxidative stress also, in agreement with other studies that measure isoprostanes (markers of damage to the cellular membranes) that decreased 2 months after RT [32]. In the case of OS in PD and HD patients still has many challenges; one of them could be focused in improving hemocompatibility of the dialysis systems; the other could be supplementation with antioxidants and modulation of the NADPH oxidase through pharmacological treatments.

In conclusion, this is the first study that evaluates the beneficial effect of RT on glycosylase after RT and shows the recovery in the natural DNA repairing capability after RT and of the antioxidants SOD and GPx measured 24 h before RT and 6 months later; these findings suggest that RT improves the conditions of altered oxidative status in ESRD patients.

## Figures and Tables

**Table 1:** Clinical and metabolic characteristics.

	24 h before RT	6 months after RT	*p*
*Clinical characteristic*			
Gender			
Male *n* (%)	35 (70)	—	—
Female *n* (%)	15 (30)	—	—
Age (years)	30.9 ± 12.0	—	—
Weight (kg)	63.9 ± 11.2	63.6 ± 10.3	0.329
PD *n* (%)	32 (64)	—	—
HD *n* (%)	18 (36)		
Catheter *n* (%)	13 (72)	—	—
Fistula *n* (%)	5 (28)	—	—

*Metabolic characteristics*			
Glucose (mg/dL)	92 ± 26	88 ± 16	0.366
Hemoglobin (g/dL)	10.8 ± 2.0	13.8 ± 1.9	<**0.0001**
Creatinine (mg/dL)	12.4 ± 4.2	1.1 ± 0.3	<**0.0001**
LDL (mg/dL)	91 ± 36	71 ± 23	**0.008**
HDL (mg/dL)	43 ± 14	52 ± 19	**<0.001**
CT (mg/dL)	162 ± 43	159 ± 31	0.439
TAG (mg/dL)	141 ± 63	180 ± 126	0.184
Albumin (mg/dL)	4 (3.3–4.4)	4.1 (4.0–4.8)	**0.008**
CRP (mg/L)	3.3 (3.0–7.4)	3.0 (3.0–4.0)	**0.003**

PD: peritoneal dialysis; HD: hemodialysis; LDL: low density lipoprotein; HDL: high density lipoprotein; TC: total cholesterol; TAG: triglycerides. CRP: C-reactive protein. The values are presented as mean and SD; albumin and CRP results are shown in median (interquartile rank).

**Table 2:** Markers of DNA status 24 h before and 6 months after RT.

	Healthy control	SEM	24 h before RT	SEM	6 months after RT	SEM	*p* WCX	*p* U-M	*p* U-M
Oxidative DNA status									
8-OHdG (ng/mL)	4.73	0.34	11.04	0.91	4.78	0.34	<0.001	<0.001	0.26
hOGG1 (ng/mL)	0.51	0.07	2.14	0.37	1.20	0.24	0.01	<0.001	0.03

Endogenous antioxidants									
SOD (UI/mL)	10.22	0.93	24.09	1.58	16.96	1.55	0.15	0.001	0.01
GPx (U/min/mg protein)	39.01	1.58	24.14	2.53	30.38	3.16	<0.001		

WCX: wilcoxon test, U-M: Mann-Whitney *U* test.
